# A Q methodology study on divergent perspectives on CRISPR-Cas9 in the Netherlands

**DOI:** 10.1186/s12910-021-00615-5

**Published:** 2021-04-26

**Authors:** Mirjam Schuijff, Menno D. T. De Jong, Anne M. Dijkstra

**Affiliations:** Department of Communication Science, University of Twente, Enschede, The Netherlands

**Keywords:** Q methodology, CRISPR-Cas9, Governance, Public perspectives, Public engagement, Human enhancement, Responsible research and innovation, RRI practice

## Abstract

**Background:**

CRISPR-Cas9, a technology enabling modification of the human genome, is developing rapidly. There have been calls for public debate to discuss its ethics, societal implications, and governance. So far, however, little is known about public attitudes on CRISPR-Cas9. This study contributes to a better understanding of public perspectives by exploring the various holistic perspectives Dutch citizens have on CRISPR-Cas9.

**Methods:**

This study used Q methodology to identify different perspectives of Dutch citizens (N = 30) on the use of CRISPR-Cas9. The Q-sort method aims at segmenting audiences based on the structural characteristics of their perspectives. Participants individually ranked 32 statements about CRISPR-Cas9 and discussed their rankings in small groups. By-person factor analysis was performed using PQMethod. Participants’ contributions to the discussions were used to further make sense of the audience segments identified.

**Results:**

Five perspectives on CRISPR-Cas9 were identified: (1) pragmatic optimism (2) concerned scepticism; (3) normative optimism; (4) enthusiastic support; and (5) benevolent generalism. Each perspective represents a unique position motivated by different ranking rationales. Sorting rationales included improving health, preventing negative impacts on society, and fear of a slippery slope. Overall, there is broad, but not universal support for medical uses of CRISPR-Cas9.

**Conclusions:**

Research on CRISPR-Cas9 should prioritise the broadly supported applications of the technology. Research and public debates on CRISPR-Cas9, its uses, its broader implications, and the governance of CRISPR-Cas9 are recommended. A discourse that includes all perspectives can contribute to the embedding of future uses of CRISPR-Cas9 in society. This study shows that Q methodology followed by group discussions enables citizens to contribute meaningfully to discourses about research.

**Supplementary Information:**

The online version contains supplementary material available at 10.1186/s12910-021-00615-5.

## Background

CRISPR-Cas9 is a relatively new technology for genetic modification. Although it offers unprecedented possibilities and has the potential of having an enormous impact on human life, little is known as yet about public attitudes toward CRISPR-Cas9. Involving citizens in the discourse about developments in technologies like CRISPR-Cas9 may have benefits for research processes, outcomes, and governance. Therefore, this study contributes to the understanding of public attitudes regarding CRISPR-Cas9. Before presenting the design and results of our study, we will first discuss CRISPR-Cas9 and the need for a public discourse on the technology.

### CRISPR-Cas9

CRISPR-Cas9 is a technique to alter the genome of organisms. It involves precise manipulations of genomic sequences specified by ‘a short stretch of guide RNA’ [1, p. 505]. Late November 2018, news broke that the Chinese scientist He Jankui claimed to have used CRISPR-Cas9 on gene CCR5 in human embryos, disabling the pathway HIV uses to infect cells, and that two apparently healthy baby girls were born [2, 3]. He intended not only to have the babies born HIV-free, but most importantly immune to ever contracting the virus, which carries great stigma in China. The work ignored the scientific consensus that the use of genetic modification on human embryos (other than in vitro for basic research purposes) is unsafe. His work therefore was not reported in a (peer-reviewed) paper, was not backed by his university, which claimed he had been on unpaid leave for months, and was heavily criticised by the scientific community. The main critique stressed that there is consensus that CRISPR-Cas9 in its current stage cannot be used for experimentation on human embryos. Besides, there is also another gene (CXCR4) that can be involved in HIV infecting cells, so the intervention only offers partial protection for the infants. In December 2019, He Jankui was sentenced to three years in prison for conducting illegal medical practices [4].

CRISPR-Cas9 can be used to correct genetic mutations that cause disease, to better understand the role of genes in diseases, and to study pharmaceutical treatment options for genetic diseases [1]. It may, theoretically, also introduce new functions in the DNA [5]. CRISPR-Cas9 is heavily studied because of ‘its high degree of fidelity, relatively simple construction and low cost’ [5, p. 1]. While CRISPR-Cas9 can be applied to any organism, our study focuses specifically on (future) human applications.

Although CRISPR-Cas9 is developing fast [6, 7], there are still problems that need to be solved before it can be used therapeutically [1]. For instance, the occurrence of unwanted mutations of genetic sequences closely resembling the targeted sequences is still problematic [1]. Other topics in need of more research are the unexpected effects of a (targeted) modification and the extent to which it is possible to reverse interventions [7].

Various ethical, legal, and societal aspects of (potential) uses of CRISPR-Cas9 for humans have been discussed, including whether and for which specific purposes somatic and/or germline modification should be allowed [5–7]. Other important aspects are how the technology may affect societal justice and equality [7] and how to balance rapid scientific discoveries, experiments on humans, translational medicine, and trust in science [6, 7]. There are also concerns related to patenting [5], the use of the technology to create organs for xenotransplantation into humans [5, 7], and the possibilities of amateur use in DIY biolabs or dual use for military (or terroristic) goals [7]. Finally, there are concerns about the governance of CRISPR-Cas9 [5, 6].

### Public discourse on CRISPR-Cas9

Several researchers and advisory boards have called for a public debate on biotechnologies [8, 9], genetic modification [10], and recently, specifically, CRISPR-Cas9 [5, 6, 11]. Public discourse on biomedical technologies like CRISPR-Cas9 is important, as these technologies are known to cause divergent reactions among experts and the public and may even lead to intractable disagreements [8]. This is illustrated by the He Jankui case described above.

To navigate morally sensitive issues in the governance of CRISPR-Cas9, it is important to have insight not only in the technology itself and its moral dilemmas, but also in the perspectives of the public. Engaging the public in decision-making about the governance of biotechnology may improve the quality of decision-making [9, 10].

So far, there have been a few surveys on public attitudes towards CRISPR-Cas9 or related technologies. Data collected in the USA showed that both somatic and germline genetic *therapy* were supported by more than half of the sample [11]. The support for somatic genetic *enhancement* was less than 50%, while only a small group supported germline genetic enhancement. Respondents generally supported the importance of public debate before the technology is applied to humans, irrespective their religious beliefs and knowledge level. In Japan, citizens generally supported (germline) genetic modification as a treatment for disease but were concerned about risks [12]. In a worldwide online survey with over 12,000 participants, 59% of the respondents agreed to the use of gene editing to cure life-threatening diseases in children or adults [13]. Another 59% agreed to the use of gene editing to cure debilitating diseases in children or adults, whereas 43% agreed to the use of gene editing for non-medical purposes. The non-medical use most supported was to boost intelligence. In an online survey among 1,013 Dutch participants focusing on reasons for standpoints in favour or against somatic genetic modification, 43 reasons in favour, 45 reasons against, and 36 conditional reasons were uncovered [14]. Many of the reasons involved emotional reactions (the ‘yuck’ and ‘wow’ factors), possible long-term consequences for society, the unnaturalness of the technology, and unacceptable health risks. In addition, there are studies on public attitudes regarding CRISPR-Cas9, gene editing, or gene modification focusing on specific applications—e. g., hereditary eye diseases [15] or non-human applications such as GM foods [16, 17].

### Study aims

Survey-based studies like those described above focus on generalised views people have on CRISPR-Cas9, aiming at indicators like percentages pro or con or average scale scores. They disregard the different overall perspectives various groups in society may have regarding CRISPR-Cas9. Meanwhile, interviews addressing complex technologies may not be able to grasp the social context and in-depth meaning for respondents and get the best understanding of their perspectives as well. Our study was designed to fill this gap. To gain a better understanding of the perspectives of citizens on CRISPR-Cas9 for the modification of the human genome, we used Q methodology to identify different segments of citizens with comparable overall perspectives on CRISPR-Cas9. The Q-sort method is a research approach specifically aiming at segmenting groups of people based on the structures of their perspectives [18–20]. Using a by-person factor analysis, Q methodology identifies *groups of participants* who make sense of, and who hence ‘sort’ a pool of items in comparable ways [21]. Because Q methodology combines quantitative and qualitative data, it allows for greater comprehensiveness of the findings than interviews or focus group discussions on its own can do. Having a better understanding of the variations in citizen perspectives on CRISPR-Cas9 can contribute to the public discourse on the technology and provide insights for the prioritisation of research on CRISPR-Cas9 and its governance. Our research aims to contribute to the public discourse on CRISPR-Cas9 [8–10]. Our research was approved by the Ethics Committee of the university.

## Method

### General overview of Q methodology

**Fig. 1:**
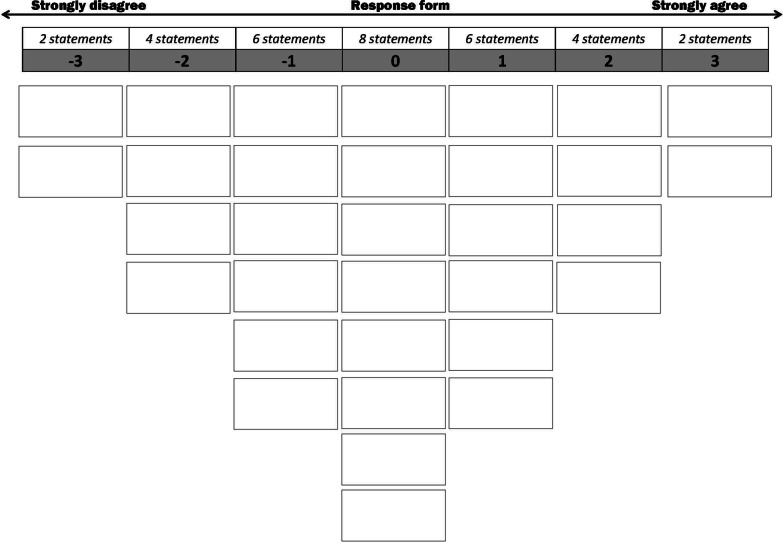
Distribution of Statements in Inverted Pyramid. Participants ranked the statements in the boxes, from ‘strongly disagree’ (left) to ‘strongly agree’ (right)

Q methodology was developed to study subjectivity and is generally seen as a research approach combining qualitative and quantitative elements [21]. Q methodology studies are often experienced as playful [20], while they let participants consider the breadth of the topic at hand. Q methodology analyses how participants rank a set of items in order to identify similar ranking patterns by means of a by-person factor analysis [19, 22]. The items must be ‘broadly representative’ of the research topic and are ranked in a forced distribution on a continuum, in our case from ‘strongly disagree’ to ‘strongly agree’ (see Fig. 1). The factors that emerge from the statistical analysis are interpreted as differential shared perspectives. The interpretation may be supplemented by participants’ motivations for their rankings as provided in interviews afterwards.

### Materials

A set of statements broadly representative of the societal aspects of CRISPR-Cas9 was developed in an iterative process [22, 23]. Statements were derived from academic literature and popular media on public attitudes and ethical, legal, and social aspects of CRISPR-Cas9. Issues were collected until the set of issues was saturated. After that, issues were clustered. The clusters addressed topics as fear for the technology, excitement, societal implications, participation, governance, use of the technology and positive and negative ethical considerations. Based on these clusters, a representative set of 32 issues covering CRISPR-Cas9 was selected and developed into statements in Dutch. All statements were as concise and clear as possible (see Additional file 1: Appendix).

The following materials were needed for each participant: the 32 statements printed on firm paper, a ‘puzzle board’ with the inverted pyramid with empty boxes to sort the statements, a recording sheet, written instructions, and a short informative text about CRISPR-Cas9. Background information was collected using a questionnaire.

### Participants

To safeguard diversity, five groups of participants were recruited in three environments: a technical university, a village, and a large city in different parts of the Netherlands. The first group consisted of academic faculty. Staff working on CRISPR-Cas9 or related genetic technologies were excluded. The other participants in the remaining four groups were citizens not affiliated to a university and not working on CRISPR-Cas9 or related genetic technologies. They were recruited via two contact persons. In total, there were 30 participants (12 female, 18 male), with ages ranging from 21 to 83 (mean 49.5). Their educational level varied from primary school to PhD.

### Procedure

Participants were invited for group sessions in three different locations across the country. All participants read and signed an informed consent form and agreed to record their session.

Sessions consisted of three parts. The first part was an introduction. The moderator explained the study goals and gave an outline of the session. After that, participants introduced themselves. At the end of the introduction, the moderator introduced CRISPR-Cas9: (1) a general text was provided to all participants and read by the moderator, and (2) a short video-clip explaining the technology was shown. The clip was taken from an educational television program for twelve-year-olds. Any questions participants had about the information on CRISPR-Cas9 or the research were answered.

In the second part, instructions for the ranking of statements were given and participants individually ranked the statements from ‘strongly disagree’ (most left on the puzzle board) to ‘strongly agree’ (most right on the board). Participants were asked to rank the statements they most strongly (dis)agreed with first. Next, working from the outside in, statements that participants (dis)agreed with less, or were neutral towards, were ranked towards the middle of the inverted pyramid (see Fig. 1) [22]. Their rankings were recorded on an answer sheet as well as photographed. For each participant, the number of the statement on the location on the puzzle board was recorded. This resulted in 30 Q sorts, or 30 rankings of the 32 statements on the inverted pyramid grid.

The third part consisted of a focus group discussion. The moderator asked all participants to share their reasons for their rankings and invited others to respond. All participants explained which statements they ranked on the extreme positions. First, the discussion focused on statements participants strongly disagreed with. Then the statements they strongly agreed with were discussed. Finally, the moderator invited the participants to bring up any statements they found important which had not been addressed. The moderator made sure every participant was included in the discussions. Participants were not allowed to change their rankings on the basis of the discussion.

Four of the five sessions took place before the aforementioned news about He Jankui’s experiment with CRISPR-Cas9 on human babies broke in late November 2018. The fifth group took place after the announcement. Although the news was widely covered by Dutch media, it did not seem to affect participants in the fifth group in the discussions.

### Analysis

Q methodology employs an inverted, or by-person, factor analysis [18, 19, 21]. This means that the participants, ranking the statements, are the variables and that ‘persons (not tests, traits or other types of variables) […] load onto the emergent factors of an inverted factor analytic study’ [21, p. 72]. Analysis was primarily based on the Q sorts while the focus group discussions served as further refinement and a check of the interpretations of the data. For the analysis, the Q sorts were intercorrelated and factor rotation was performed [22] using the PQMethod software. Initially seven factors were extracted, two of which were excluded because their eigenvalue was below 1.00 (the Kaiser-Guttman criterion). The five remaining factors were rotated using varimax rotation. The five accepted factors explained 63% of the study variance. Factor loadings of 0.48 or above were considered significant at the *p* < 0.01 level [22]. Of the 30 rankings by the participants, 26 loaded significantly on one of the five factors, two were confounded (loading significantly on more than one factor), and two did not have significant factor loadings. Each factor represents a shared perspective of a group of participants, but participants who load significantly on one factor will not have ranked the statements exactly the same. Weighted averaging was used to calculate the ideal–typical Q sort for each factor—called the factor arrays (see Additional file 1: Appendix) [22].

Next, the five factors were interpreted by a holistic inspection of the factor arrays. To understand and explain the perspective expressed in the factor arrays, for each factor the highest and lowest ranking statements, statements that were ranked significantly higher or lower than in other factors, and any other noteworthy statement rankings were inspected [22].

The next step involved assessing the uniqueness of each factor. Statements about similar aspects or topics of the discourse on CRISPR-Cas9 were clustered. The scores of the five factors on these clusters were then analysed in order to identify relevant rationales of each factor as well as the relative differences between the five factors. Special attention was given to the statements that were ranked at either extreme of the inverted pyramid, clusters of statements that distinguished a factor from the others, as well as individual statements that were scored differently than the other statements in a factor. This allowed us to present the factor narratives and their different emphases better.

Statements ranked towards the middle of the inverted pyramid were given careful consideration in the interpretation. The forced distribution of the statements implies that rankings towards the middle do not necessarily reflect ‘slightly disagree’, ‘neutral’, or ‘slightly agree’; because the participants had only a limited number of positions to rank the statements they most (dis)agreed with (see Fig. 1).

The group discussions were transcribed and analysed to further support the interpretation of the five factors. For each factor, the explanations and remarks of participants whose Q sort loaded positively on that factor were collected and studied. Demographic information was considered in this analysis as well. The factors were labelled to succinctly summarise their sorting rationales.

## Results

Q methodology helped participants to parse their thoughts about a complex—and to some participants new—topic before deliberations with other participants. Ranking the statements was experienced as fun and useful by the participants. When asked if they had missed any statements during the ranking, no statements were suggested by participants. The five resulting factors are presented below. The factor narratives are presented in order of their eigenvalue, variance, and the number of participants with positive factor loadings. The narratives of the two factors on which most participants loaded and that explained most of the variance are presented in full. The factors that explained less of the variance (factors 3, 4, and 5) are presented in a more condensed way. While for each factor at least two Q sorts are significantly associated with this factor, it is important to note that the narratives are based on the ‘ideal typical’ weighted average of the factor as presented in the Additional file 1: Appendix and not on any individual participants’ ranking. In all narratives, emphasis is placed on the (clusters of) statements that contribute most to understanding the unique perspective of the factors. An overview of the rationales of the factors is given in Table 1. An overview of all statements and factor loadings can be found in the Additional file 1: Appendix. The statements and their rankings on the factor arrays are presented as (statement number at assigned ranking).

**Table 1:** Overview of the Five Factors Based on the Q-Sorts

Factor	Label	Ranking rationale(s)
1	Pragmatic optimism	The medical benefits of CRISPR-Cas9 are supported, unlike non-medical uses. Moral, societal, and governance considerations are less important
2	Concerned scepticism	CRISPR-Cas9 has merit as a scientific development, but its applications on humans should be limited. There are many fundamental concerns around the development and use of CRISPR-Cas9
3	Normative optimism	CRISPR-Cas9 offers medical benefits, but only if moral boundaries are safeguarded and societal side-effects are mitigated
4	Enthusiastic support	CRISPR-Cas9 is a positive development that improves healthcare. There are few fundamental concerns
5	Benevolent generalism	CRISPR-Cas9 is a fascinating, yet complex scientific development. Governance and public participation are important, and other issues will be managed as they arise

There were two consensus statements, which did not differ significantly among the factors: (1) ‘CRISPR-Cas9 raises too many questions to use the technology,’ and (2) ‘Further development of CRISPR-Cas9 justifies using embryos’. Both statements did not correspond to unique aspects of any of the factors.

With only one exception, no correlations were identified between the factors and participants’ background characteristics. The two participants in factor 4 (the most liberal view on CRISPR-Cas9) described themselves as non-religious and politically liberal.

### Factor 1: Pragmatic optimism

This factor had an eigenvalue of 7.8 and explained 26% of the variance. Fourteen participants were significantly associated with this factor. Six participants were scientists, four were retirees, two worked in the for-profit sector, one worked for the government, and one was a student. They described themselves as hardly or not religious. Most participants were moderately interested in politics (some not at all). They kept up to date on technological developments by following media (mostly the news, documentaries or other tv programmes, newspapers, or the internet). Participants in this factor focused on medical benefits of CRISPR-Cas9. Non-medical uses were not supported. Moral, societal, and governance considerations were less important.

In this factor, trust in the medical usage of CRISPR-Cas9 was central: Statements on preventing and curing hereditary diseases were ranked either highest or second highest (11 at 3; 13 and 26 at 2). The statement that it is good that CRISPR-Cas9 improves the lives of people with hereditary diseases was also ranked high (32 at 3). This indicates that the contribution of CRISPR-Cas9 to the health and quality of life of people with hereditary diseases was an important sorting rationale in this perspective.

More on the long term, a better understanding of our DNA as a result of CRISPR-Cas9 was also ranked high (28 at 2). Nevertheless, in this perspective enthusiasm for the technology (7 and 5 at 1) did not entail that all uses of CRISPR-Cas9 were supported. The technology should not be used to modify DNA at will (15 at − 2). It should always be used voluntarily; as it would be undesirable if DNA modification became the norm in society (31 at − 2). Two participants explained their reasoning:

I would only use it for purely medical purposes, and not for anything else…. The technology is developed with the best intentions in mind, but at a certain point it will be applied to non-medical conditions that will allow you, well, if you can afford it, to increase your quality of life a lot, have smarter children, live longer, and you’ll see that others cannot afford it. And that seems very undesirable to me. [P04]

I think, I see good things in CRISPR-Cas9, as long as it is regulated strictly and is used for certain, for example hereditary, diseases. Not for when you want blue eyes, blond hair, or whatever, that kind of things, those are unacceptable to me. And I would not want them in the future either. That is why I think that, if we regulate it strictly now, you may be able to prevent that it will develop like that. [P02]

Moral and societal considerations were not ranked very prominent in this factor, which points to a pragmatic perspective valuing benefits over moral concerns or societal implications. Moral statements expressing that the CRISPR-Cas9 is ‘wrong’, ‘should never be used’, and is ‘unnatural and therefore unwanted’ were ranked low (3 and 9 at − 3; 27 at − 2). Most statements on societal implications of CRISPR-Cas9 were ranked at either 1 or − 1, suggesting that there were concerns about CRISPR-Cas9, but other considerations were more pressing. There appeared to be some fear of increasingly extreme uses (18 at 1). There was also some fear that CRISPR-Cas9 will eventually overburden the healthcare system (16 at 1) and that it will only be available for the rich (14 at 1). One participant explained in the focus group that there had been differences in healthcare insurances in the past: People with private insurance received faster and better care than people with mutual insurance. She feared a return to this situation with the introduction of new technologies like CRISPR-Cas9:

In the past, there was a class divide in healthcare, and it is a good thing that this no longer exists. This situation should never come back, in my opinion…. When I was a child, we had premium healthcare insurance, which meant that you could sit in a special waiting room at the doctor's office and were helped faster…. It really was not nice, even though we had premium healthcare insurance ourselves. You do not want to divide people into two groups and treat one group better. [P19]

Regarding governance of CRISPR-Cas9, this factor showed mixed results. In the factor array, governance was not prominent. Strict regulation (10 at 1) is ranked lower than independent monitoring of the technology (12 at 2). In the focus group, however, governance was emphasised more. One participant said: ‘Regulation is a safeguard to me’. Furthermore, the need for global governance was brought up by the participants in several discussions. One participant feared that medical tourism will otherwise arise:

I am afraid it will be a slippery slope that will end in creating designer babies. That is why I think independent oversight and strict regulations of the conditions when it can be used [are important]. And I prefer them to be global, because otherwise people will just travel to Malaysia for it. [P15]

The parties involved in the governance of CRISPR-Cas9 were less important in this factor. Scientists and citizens can play a role (4 and 8 at 0); politicians (6 at − 1) and especially (pharmaceutical) companies (2 at − 2) receive less support.

Statements about fears and future developments received moderate rankings in this factor. Participants did not see CRISPR-Cas9 as a scary technology (1 at − 1), were neutral about the statements that that there are still too many unanswered questions (17 at 0) and that CRISPR-Cas9 may be abused (19 at 0), and had moderate rankings of risks (21 at − 1) and the tolerability of using embryos (22 at − 1) and animal experiments (24 at 0) to develop the technology further.

### Factor 2: Concerned scepticism

This factor had an eigenvalue of 4.8 and explained 16% of the variance. Five participants loaded significantly on this factor: Three came from scientists, two from non-scientists. Three were male, two female. Three described themselves as non-religious, one was moderately religious and one believed in reincarnation. They considered themselves to be low to moderately interested in politics. They kept up to date on technological development by following the news and online media. In this perspective, CRISPR-Cas9 has merit as a scientific development, but its applications on humans should be limited. Many moral and societal considerations surround the development and use of CRISPR-Cas9.

Governance of CRISPR-Cas9 was very important in this perspective, which manifested itself in high rankings of statements related to participation and regulation. The statements that strict regulation of CRISPR-Cas9 is important and the statement that there is a need for independent oversight (10 and 12 at 3) both were ranked highest. One participant stressed the importance of governance and clear regulations as follows:

That there is at the very least consensus about where it can be used, what is allowed, what is not allowed. I think, if you say something is not allowed, that is always strict for scientists and for companies who can do it. But well, it is not allowed, and it can be done, well, what is strict in a situation like that? You cannot only allow half. You also do not ride a bike half. [P01]

This perspective also involved specific preferences for whom should participate in determining the future of CRISPR-Cas9. Participants had considerably more trust in scientists (4 at 2) than in politicians (6 at − 2) and, particularly, citizens and companies (8 and 2 at − 3).

In this perspective, some of the moral considerations were more salient than others. Although it was seen as positive that CRISPR-Cas9 contributes to a better understanding of our DNA (28 at 2) and improves the lives of people with hereditary diseases (32 at 1), in this factor people should not play God (25 at 2), that our DNA becoming modifiable is not a good development (2 at − 2), that CRISPR-Cas9 is unnatural and therefore unwanted (27 at 1), and that modification of DNA must certainly not be obligatory (31 at − 2). Still, participants with this perspective would not qualify CRISPR-Cas9 as wrong (3 at − 1) and were moderately positive about the possibility to make organs of animals suited for transplantation into humans (30 at 1). Together, these rankings suggest that in this perspective CRISPR-Cas9 is seen as a valuable research approach, but one that should largely be confined to petri dishes in the laboratory. Unconditional use on human beings was not supported.

The interpretation that in this perspective CRISPR-Cas9 is valued mostly as a research tool was supported by the rankings of the statements related to its use. Treatment and prevention of hereditary diseases were both ranked neutrally (11 and 13 at 0). In the discussion, some participants expressed some support of treatments with CRISPR-Cas9, indicating that the neutral ranking can suggest tentative support rather than neutrality.

If you ask me, it should never be used. It will become more and more extreme if you go down that road, if we go down that road. Then it will never stop. [P17]

I found it difficult, because I do believe the technology should be used for hereditary things, to correct them. So, when you have people who have hereditary, like, something, but then can have children who have better chances, I would approve of that. But on the other hand, you have the point about embryos and that is… You are moving in the direction of human enhancement and then you’ll have healthy embryos that you are going to give certain characteristics that they want to, or don’t want to, give to their offspring. That goes too far. [P01]

I disagree that people should be able to modify their DNA as they like, because I think, well, life is also to a certain extent a given. [P06]

Several types of considerations appeared to be less important in this perspective. Statements related to the development of CRISPR-Cas9 were ranked low to neutral: In this perspective it would not be an acceptable risk to alter wrong sections of DNA (21 at − 1). The use of laboratory animals or embryos to further develop the technology was also not supported (24 and 22 at − 1).

Potential societal implications of CRISPR-Cas9 also were ranked around the ‘neutral’ position in this factor. The implication ranked highest was that people can become genetically more identical (23 at 1). The statement about CRISPR-Cas9 becoming a luxury for the rich was ranked neutrally (14 at 0), as were the statements about the potential burden on the healthcare system and increasing inequalities in society (16 and 20 at 0). This does not imply that such considerations were not important, but rather that other types of considerations outweighed them in the ranking of statements. In the focus groups, one participant, for example, brought up a concern about increasing corrosion of solidarity principle in the healthcare system:

It is going in the direction like when you have lung cancer and you are a smoker, then it is your own fault and you’re on your own. It is developing in that direction. So, when you do not intervene in your own DNA, then is will be your fault if you’re sick, and you’ll be on your own. [P17]

Finally, affect only played a minor role in this perspective. While there was some agreement that CRISPR-Cas9 offers great possibilities (7 at 1), it was not seen as exciting (5 at − 1) or scary (1 at 0). This minor role can be explained by the emphasis on governance and the focus on CRISPR-Cas9 as a research approach, about which still too much is unknown to consider using it (17 at 2).

### Factor 3: Normative optimism

This factor had an eigenvalue of 2.7 and explained 9% of the variance. All three participants that loaded significantly on this factor were retirees, and two of them were female. Two participants lived in a city, one in a village. Their educational levels were (prolonged) primary school, high school, and higher vocational education. They described themselves as non-religious, moderately religious, or only raised religiously. Two were moderately interested in politics; one not interested. They informed themselves on technological developments by the traditional media and literature. In this perspective, CRISPR-Cas9 may offer medical benefits, but only if moral boundaries are safeguarded and societal side-effects are mitigated.

In this factor, moral and societal considerations regarding CRISPR-Cas9 were very important. The statement that humans should not play God was the moral consideration with the highest ranking (25 at 3). Furthermore, people should not be obliged to modify their DNA, nor should non-medical modifications be possible (31 and 15 at − 3). These rankings indicated strong moral boundaries regarding the possible uses of CRISPR-Cas9. The most highly ranked reason was curing people with hereditary diseases (11 at 2), which implies that the moral considerations did not result in a categorical rejection of use on humans.

The governance of CRISPR-Cas9 was very important in this perspective. Independent monitoring of the technology was ranked highest (12 at 3) and higher than the statement on strict regulation (10 at 1), suggesting that monitoring was more important than strict regulation. The desire to have independent oversight indicates a need for checks and balances on a technology that evokes strong moral objections. Regarding the parties participating in deciding about the future of CRISPR-Cas9, participants in this factor felt more comfortable with scientists (4 at 0) and politicians (6 at − 1) than with companies and citizens (2 and 8 at − 2). Companies are ruled by financial incentives rather than by what is right, which participants feared would lead to wrong usage of the technology. However, the rankings suggest that it is more important that there are rules and monitoring than who should have a say on its future.

Societal considerations were also important in this perspective. CRISPR-Cas9 should be available to everybody and not be a luxury only the rich can afford (14 at 2). Yet, it should not become too great a financial burden for the healthcare system (16 at 2). A third concern was that CRISPR-Cas9 could lead to people becoming more genetically identical (23 at 2). The statement about the risks of increasing inequality in society, however, was ranked neutrally (20 at 0). Together, these rankings indicate concerns that the introduction of CRISPR-Cas9 within society may have undesirable effects. This is in line with the attention for governance of the technology.

Despite emphasising the moral, societal, and governance aspects of CRISPR-Cas9, participants in this factor did not reject the use of the technology. They agreed to an extent with the statement that it is good that CRISPR-Cas9 can improve the lives of people with hereditary diseases (32 at 1), as well as with other statements about uses. Curing people with hereditary conditions was the most important use of the technology (11 at 2). Prevention of hereditary diseases and modifying embryos to prevent passing down hereditary conditions to future generations were also supported (13 and 26 at 1). Xenotransplantation was ranked low in this perspective (30 at − 2) as was the use of CRISPR-Cas9 to alter DNA as desired (15 at − 3), indicating that this factor clearly differentiated between good (medical) and bad (non-medical) uses of CRISPR-Cas9.

The feelings CRISPR-Cas9 evokes were less important. The technology was not scary (1 at − 2) and even somewhat exciting (5 at 1). Yet, CRISPR-Cas9 is not seen to offer great possibilities (7 at − 1). In the light of the emphasis on moral considerations and the clear distinction between good and bad uses of CRISPR-Cas9, these rankings make sense. Some moral statements also were not as relevant to the ranking rationale of this perspective, such as ‘CRISPR-Cas9 is wrong’ or the statement that the technology is unnatural and therefore unwanted (3 and 27 at 0). In the words of one participant:

Of course it is unnatural, but on the other hand… I believe, sometimes you have to do something that is unnatural. [P28]

What is acceptable for the further development of CRISPR-Cas9 was unclear in this perspective, although the risk of modifying wrong pieces of DNA and testing the technology on animals including primates were not supported (21 and 24 at − 1).

### Factor 4: Enthusiastic support

This factor had an eigenvalue of 1.8 and explained 6% of the variance. Two participants were significantly associated with this factor: one was a scientist and one a retiree. Both participants were male and described themselves as non-religious. Their political orientation was liberal, although only one o considered himself to be interested in politics. The participants in this factor were outright positive about the health improvements CRISPR-Cas9 promises and did not see many fundamental concerns.

The participants embraced the possibilities CRISPR-Cas9 offers. The technology was absolutely not seen as scary (1 at − 3). It can have positive effects, such as improving the lives of people with hereditary diseases is (32 at 2) and gaining a better understanding of our DNA (28 at 2). The participants strongly disagreed that the technology should never be used (9 at − 2) and playing God was not a concern (25 at − 2). This perspective was in fact so positive about CRISPR-Cas9 that the participants saw using CRISPR-Cas9 to improve DNA as an obligation or ‘moral duty’ (31 at 3). These statements suggest optimism towards CRISPR-Cas9. One participant stated:

If it really is a promising technology and people can benefit from it, then I do not understand why it should never be used. [P08]

Participants in this factor strongly believed that further development and use of CRISPR-Cas9 is a good cause. They found it an acceptable to risk that wrong pieces of DNA may be modified in the further development of the technology (21 at 2). This also reflects in the neutral ranking of the statement that there are too many unanswered questions to proceed using the technology (17 at 0). Experiments were seen as a way to learn about DNA and the use of CRISPR-Cas9 itself. In this perspective, using animals including primates to develop CRISPR-Cas9 received a lot of support (24 at 3), but the use of embryos in tests was ranked neutral (22 at 0). Discussions did not clarify why the use of animals received much more support than the use of embryos.

Regarding governance and participation in determining the future of CRISPR-Cas9, participants in this factor found independent oversight slightly more important than strict regulations (12 at 1 vs. 10 at 0). Scientists were trusted most for having responsibility to determine the future of the technology (4 at 1). The participants had less confidence in companies, citizens (2 and 8 at − 1), and especially politicians (6 at − 3). The trust in scientists may be attributed to their understanding of the technology and their lack of financial or political interests in its future.

The participants were less outspoken about uses of CRISPR-Cas9. Modifying embryos to prevent passing down hereditary conditions was ranked higher than curing or preventing hereditary conditions in individuals (26 at 2; 11 and 13 at 1). Xenotransplantation was ranked relatively high (30 at 1). The ranking pattern suggests that the specific applications were not so important, and that the overall goal of improving lives prevailed. Altering DNA as desired received a neutral ranking (15 at 0).

The impact CRISPR-Cas9 may have on society was not very important in this perspective. This can be inferred from the moderate rankings of statements about the technology being a luxury only the rich can afford (14 at − 1), the financial impact on the healthcare system (16 at 0), and people becoming genetically more identical (23 at 0). Only the statement that further development of CRISPR-Cas9 would lead to increasing inequality in society received a more outspoken ranking, indicating disagreement (20 at − 2).

Ratio rather than emotion appears to inform the ranking rationale in this factor. Statements expressing that CRISPR-Cas9 is scary or exiting or that it has great possibilities received low rankings (1 at − 3, 5 and 7 at 0). This does not mean that the sorting pattern was free from emotion, but rather that it was less important for the participants in expressing themselves.

### Factor 5: Benevolent generalism

This factor also had an eigenvalue of 1.8 and explained 6% of the variance. Two participants were significantly associated with this factor: one was a PhD student and one a retiree. Both were male. One was religious, the other not. One was involved in politics, the other was not interested in politics. Apart from their Q sort, they did not appear to have many similarities. In this perspective, CRISPR-Cas9 is seen as a fascinating, yet complex scientific development. It is too early to know anything for sure, except that governance and public participation are important.

The participants saw CRISPR-Cas9 as an exciting technology (5 at 3) that can lead to great things (7 at 2). But it is also a little scary (1 at 1). Despite considering the technology the scariest of all five perspectives, the participants in this factor did not believe CRISPR-Cas9 should never be used (9 at − 3). Fear should thus not stand in the way of excitement or using the possibilities the technology offers. CRISPR-Cas9 thus evoked mixed emotions in this perspective, which could also be seen in the ranking of the other statements.

Independent monitoring of CRISPR-Cas9 was very important (12 at 3). The technology should be regulated strictly (10 at 2) and it mattered to the participants who are involved in determining the future of CRISPR-Cas9: Citizens were seen as the most suited to determine this future (8 at 2); the other groups—companies and politicians (2 and 6 at − 1) and scientists (4 at − 2)—were ranked much lower. These rankings indicate that it was important to them that normal citizens have a say in determining the future of the technology. This interpretation is corroborated by the claim of one of the participants that he is always involved in political and societal issues.

With regard to moral considerations, this perspective does not believe that CRISPR-Cas9 is wrong (3 at − 2), nor that the technology should never be used (9 at − 3). In this perspective, people are not obliged to improve their DNA using CRISPR-Cas9 (31 at − 2), nor can the technology be used at will.

I think that on the whole medical technologies cannot be used at liberty, so CRISPR-Cas9 should be treated likewise. [P09]

With the exception of ensuring that CRISPR-Cas9 will not become a luxury only available to the rich (14 at 2), most statements related to the development of CRISPR-Cas9, its applications, its possible societal impact, and the remaining moral considerations were all ranked around the centre. This indicates that the rationale of this perspective focuses on realising the ‘great possibilities’ of CRISPR-Cas9 (7 at 2), with governance and citizens participation as important safeguards. Questions or issues related to development, use, or societal consequences can then be dealt with when they arise.

## Discussion

### Main findings and implications

The aim of this Q methodology study was to explore the perspectives of Dutch citizens on CRISPR-Cas9 in a comprehensive manner. While it is not the goal of Q methodology to compare the perspectives [22], the five factors differ with respect to the uses they see for CRISPR-Cas9, what matters most to them about CRISPR-Cas9, and the ranking rationales (see Table 1). In the perspective of pragmatic optimism there is (only) support for the medical uses of CRISPR-Cas9 to increase health and well-being. In concerned scepticism, CRISPR-Cas9 is viewed as a scientific development that should have limited applications directly on humans, as there are many fundamental concerns. In the normative optimism perspective CRISPR-Cas9 offers medical benefits, but only if moral concerns and negative societal effects are mitigated. In the perspective of enthusiastic support CRISPR-Cas9 is seen as a positive development and no real fundamental concerns exist. Finally, in the perspective of benevolent generalism, participants are fascinated by CRISPR-Cas9, believing that possible risks can be managed when they arise as long as there is a good governance structure with public participation. There is some agreement between (some of) the perspectives, but each factor represents a unique point of view.

While there are different perspectives on CRISPR-Cas9, there is broad—although not universal—support for the use of the technology for the treatment and prevention of hereditary conditions. Concerned scepticism is found to only support the use of CRISPR-Cas9 as a research tool, which can lead to more knowledge and thus indirectly improve treatment and prevention of genetic diseases or via xenotransplantation. Enhancement reasons are less broadly supported. Except for enthusiastic support, most perspectives disagree with enhancement uses of CRISPR-Cas9. And even in the enthusiastic support perspective CRISPR-Cas9 for enhancement was only ranked neutrally. The negative attitude toward enhancement uses aligns with earlier findings [11, 13], although acceptance of these technologies might rise over time [24, 25]. Together, the findings on medical and non-medical uses of CRISPR-Cas9 imply that there currently is broad support for research on and medical use of CRISPR-Cas9.

In the perspective of concerned scepticism there are fundamental concerns about the ethical and societal implications of CRISPR-Cas9 and direct use of the technology on human beings is not supported, with a remarkable exception for xenotransplantation. The other perspectives ranked direct use of the technology for treatment and prevention higher than that of concerned scepticism but participants generally were less positive about xenotransplantation. Only the enthusiastic support group ranked the statement on xenotransplantation relatively high. Earlier studies on public attitudes in the Netherlands showed that people were generally unfamiliar with xenotransplantation and that it was considered very risky by a large majority [26]. Xenotransplantation hardly played a role in the discussions, which could mean that participants are still unfamiliar with that possibility or that public attitudes on this topic are becoming more positive.

Our findings emphasise the importance of gaining a better understanding of how citizens relate to innovative technologies. In debates about the future development of CRISPR-Cas9, attention should be given to include all perspectives, which are so divergent that agreement about the future of CRISPR-Cas9 seems unlikely. Giving each perspective equal respect will improve the quality of decision making [8–10]. Even if decisions do not satisfy their views, people are more likely to accept them if they experienced respect during the decision-making process [10]. A discourse on CRISPR-Cas9 that is open to all perspectives and allows participants to express their views is recommended to embed future uses of CRISPR-Cas9 in society.

More in general, discourses about innovative technologies do take place within their larger social and political contexts, and people form their perspectives not only on direct interactions with science but often in a mediated way within that socio-political context [27]. Understanding these dynamics when communicating about innovative technologies is important, and social sciences research as exemplified with the Q methodology can contribute to such democratic deliberation. In that sense, potentially transformative technologies like CRISPR-Cas9 underline the need for nuanced deliberations which can deal with different perspectives [28].

The perspectives identified in our study are holistic. This is a logical consequence of the Q methodology used, which centres around a set of items that are a broad representation of the discourse on a topic [19, 22]. Too narrow a focus on risk–benefit thinking has been critiqued for neglecting broader aspects [9, 29]. Our findings as well as our participants’ enthusiasm showcase that citizens can meaningfully contribute to discourse on science and technology and are eager to do so. The willingness of the participants to contribute, the identified holistic perspectives, and the open exchange of perspectives underscore the importance of responsible research and innovation (RRI) initiatives, which entail ‘taking care of the future through collective stewardship of science and innovation in the present’ [30, p. 1570]. RRI has become increasingly central in European policy making and academic work [31–33]. RRI can be integrated in academic practices in different ways. Combining the Q methodology with group discussions integrates two types of RRI practices, namely opening up and anticipating the ethical, legal, and social implications of research [34].

### Limitations

The present study has several limitations that need to be kept in mind. The Q methodology aims at exploring perspectives and does not generate overall scores that can be generalised to the general public. Although the statements were designed as concise and clear as possible, in retrospect some contained ambiguities (e.g. 19). This may have affected how the participants interpreted them. Furthermore, the study was held in one country, which means that the findings might not apply to other countries and cultures. And finally, the technology studied and the public discourse on the technology are still in their early stages, which means that perspectives may change over time as both the technology and the discourse mature.

### Suggestions for future research

Both the technology and the public discourse on CRISPR-Cas9 are in the early stages of development. This means that there are many open questions. Important research questions include a better understanding of citizens’ perspectives on CRISPR-Cas9, its uses, and embedding in society in relation to the array of hereditary conditions it could be used for. There is already some research on this [15], but more research is needed for a better understanding. In particular research could delve deeper in the way citizens make sense of the technology, how they process new information about the latest developments—breakthroughs or crises—and how they can contribute to the technology in co-creation processes. International comparisons, particularly with other cultures, would also be a fruitful direction for research. It would be interesting to see whether out typology of perspectives would be corroborated in other national contexts. Likewise, it would be interesting to see whether a similar typology of perspectives emerges in the context of other radical technological innovations, such as nanotechnology or artificial intelligence.

Furthermore, there are many hereditary conditions, which differ, among other things, in how severely they affect health and well-being. Our study did not explicitly differentiate between severe and less severe hereditary conditions or on the weighing of treatment versus prevention in specific situations. A stronger focus on such dilemmas would be an interesting line of future research as well.

### Conclusion

In this study, we used Q methodology to explore the various perspectives citizens may have on CRISP-Cas9. Participants were asked to rank the extent to which they agreed with statements about various aspects of the technology. The analysis focused on distinguishing different groups with similar holistic perspectives on CRISPR-Cas9. Our analysis resulted in five perspectives on CRISPR-Cas9, which we labelled as pragmatic optimists, concerned sceptics, normative optimists, enthusiastic supporters, and benevolent generalists. The five perspectives have in common that the participants managed to come up with mixtures of appreciation and concerns, in different shades and on different aspect. The typology that emerged can be seen as an intermediate contribution between highly individual interview results and generalized survey findings. It can help to better understand the perspectives from which citizens gather knowledge and form opinions about CRISPR-Cas9.

## Supplementary Information

**Additional file 1: Appendix**. Statements and rankings in Factor Arrays.

## Data Availability

All Q study data generated and analysed during this study are included in this article. All transcripts are available from the corresponding author upon reasonable request.
