# Supplementary material for: A Q methodology study on divergent perspectives on CRISPR-Cas9 in the Netherlands
Source: BMC Med Ethics. 2021 Apr 26;22:48. doi: 10.1186/s12910-021-00615-5 (PMC8074506; doi:10.1186/s12910-021-00615-5)
Supplement: Supplementary file 1 — Additional file 1: Appendix. Statements and rankings in Factor Arrays. [file 12910_2021_615_MOESM1_ESM.docx]

**A Q methodology study on divergent perspectives on CRISPR-Cas9 in the Netherlands**

Mirjam Schuijff

Department of Communication Sciences, University of Twente

Menno D.T. de Jong

Department of Communication Sciences, University of Twente

Anne M. Dijkstra (corresponding author)

Department of Communication Sciences, University of Twente

a.m.dijkstra@utwente.nl

**Additional file 1: Appendix: Statements and Rankings in Factor Arrays**

|  | Statement | Factor 1 | Factor 2 | Factor 3 | Factor 4 | Factor 5 |
| --- | --- | --- | --- | --- | --- | --- |
| 1 | CRISPR-Cas9 is scary | -1 | 0 | -2 | -3* | 1 |
| 2 | Companies are best suited to determine the future of CRISPR-Cas9 | -2 | -3 | -2 | -1 | -1 |
| 3 | CRISPR-Cas9 is wrong | -3 | -1 | 0 | -1 | -2 |
| 4 | Scientists are best suited to determine the future of CRISPR-Cas9 | 0 | 2 | 0 | 1 | -2 |
| 5 | CRISPR-Cas9 is an exciting new technology | 1 | -1 | 1 | 0 | 3* |
| 6 | Politicians are best suited to determine the future of CRISPR-Cas9 | -1 | -2 | -1 | -3 | -1 |
| 7 | CRISPR-Cas9 offers great possibilities | 1 | 1 | -1 | 0 | 2* |
| 8 | Citizens/civilians are best suited to determine the future of CRISPR-Cas9 | 0* | -3 | -2 | -1 | 2* |
| 9 | CRISPR-Cas9 should never be used | -3 | 0 | -1 | -2 | -3 |
| 10 | CRISPR-Cas9 should be regulated strictly | 1 | 3 | 1 | 0 | 2 |
| 11 | CRISPR-Cas9 should be used to cure hereditary diseases | 3 | 0 | 2 | 1 | 1 |
| 12 | CRISPR-Cas9 requires independent oversight/regulation | 2 | 3 | 3 | 1 | 3 |
| 13 | CRISPR-Cas9 should be used to prevent hereditary diseases | 2 | 0 | 1 | 1 | 1 |
| 14 | CRISPR-Cas9 should not be a luxury available to the rich only, but should be accessible to everybody | 1* | 0 | 2* | -1 | -2 |
| 15 | CRISPR-Cas9 should be used to alter DNA as desired | -2 | -2 | -3 | 0 | 0 |
| 16 | CRISPR-Cas9 should not overburden the healthcare system financially | 1 | 0 | 2 | 0 | 1 |
| 17 | CRISPR-Cas9 raises too many questions to use the technology** | 0 | 2 | 0 | 0 | 0 |
| 18 | Further development of CRISPR-Cas9 leads to increasingly more extreme applications | 1 | 0 | 0 | -2 | -3 |
| 19 | People can abuse CRISPR-Cas9 since it’s relatively easy to use | 0 | 1 | -1 | -1 | -1 |
| 20 | Further development of CRISPR-Cas9 leads to increasing inequality in society | -1 | 0 | 0 | -2* | 0 |
| 21 | It is an acceptable risk that CRISPR-Cas9 might alter wrong sections of DNA | -1 | -1 | -1 | 2* | 0 |
| 22 | Further development of CRISPR-Cas9 justifies using embryos** | -1 | -1 | 0 | 0 | -1 |
| 23 | CRISPR-Cas9 can lead to people becoming genetically more identical | 0 | 1 | 2 | 0 | 1 |
| 24 | Further development of CRISPR-Cas9 justifies using animals like chimpanzees in experiments | 0 | -1 | -1 | 3* | -1 |
| 25 | People should not play god by using CRISPR-Cas9 | -1 | 2 | 3 | -2 | -1 |
| 26 | It is good that CRISPR-Cas9 can modify embryos so hereditary diseases will not be passed on | 2 | -1* | 1 | 2 | 2 |
| 27 | CRISPR-Cas9 is unnatural and therefore unwanted | -2 | 1 | 0 | -1 | 0 |
| 28 | It is good that CRISPR-Cas9 increases our knowledge of our DNA | 2 | 2 | 1 | 2 | 0* |
| 29 | It is a good development that CRISPR-Cas9 makes our DNA modifiable | 0 | -2* | 0 | 1 | 1 |
| 30 | It is a good cause that CRISPR-Cas9 can make animals’ organs suitable for transplantation into humans | 0 | 1 | -2* | 1 | 0 |
| 31 | People are obligated to improve their DNA with CRISPR-Cas9 | -2 | -2 | -3 | 3* | -2 |
| 32 | It is a good cause that CRISPR-Cas9 improves the lives of people with hereditary diseases | 3 | 1 | 1 | 2 | 0 |

Note: Statements are translated from Dutch by the authors. Scores with an * indicate a distinguishing statement for this factor (p < .05). Statements marked with ** are consensus statements.
